# Thyroid-associated ophthalmopathy: Using diffusion tensor imaging to evaluate visual pathway microstructural changes

**DOI:** 10.3389/fneur.2022.1025666

**Published:** 2022-11-02

**Authors:** Rui Li, Jing Li, Zhenchang Wang

**Affiliations:** Department of Radiology, Beijing Friendship Hospital, Capital Medical University, Beijing, China

**Keywords:** thyroid associated ophthalmopathy, lateral geniculate, optic radiation, diffusion tensor imaging, arterial spin labeling

## Abstract

**Objective:**

The aim of this study was to determine whether the visual pathway is affected by thyroid-associated ophthalmopathy (TAO) before the development of dysthyroid optic neuropathy (DON) with diffusion tensor imaging (DTI).

**Materials and methods:**

Fifty-seven TAO patients (22 mild, 35 moderate-severe TAO) and 30 healthy controls (HCs) were included. The DTI parameters of the lateral geniculate (LG) and optic radiation (OR) were measured. A full ophthalmic examination such as intraocular pressure, exophthalmos, and visual acuity was performed. Clinical activity scores (CAS) were also calculated. One-way ANOVA and Pearson's correlation were carried out. A binary logistic regression was used to identify variables that can diagnose TAO.

**Results:**

Moderate-severe TAO patients showed significantly lower fractional anisotropy (FA) and higher mean diffusivity (MD) than HCs for both LG and OR (*p* < 0.05). FA of OR was negatively correlated with CAS and intraocular pressure (*p* < 0.05). Multivariate analysis showed that FA of LG and FA of OR were a significant predictor for the diagnosis TAO.

**Conclusion:**

Diffusion tensor imaging parameters of the visual pathway were significantly altered in moderate-severe TAO patients. The FA of LG, in particular, can be used as a sensitive imaging biomarker for assessing subclinical visual pathway damage in TAO.

## Introduction

Thyroid-associated ophthalmopathy (TAO) is the most prevalent orbital disease worldwide and produces severe aesthetic and functional impairment. It usually impacts patients' quality of life, affecting self-esteem, relationships, and daily activities. TAO patients frequently complain of photophobia, excess tearing, and grittiness. The most severe ophthalmic complication of TAO is dysthyroid optic neuropathy (DON), which affects 4–8% of patients (1). The exact pathogenesis of DON is unclear. Optic neuritis, compression, or stretch may be potential DON mechanisms (2). A permanent and irreversible loss of vision may result from untreated DON. The early detection of visual pathways changes in TAO patients is necessary to avoid vision loss.

Recently, along with the development of magnetic resonance imaging (MRI) techniques, a few studies have investigated the dysfunction of optic nerve in TAO cohort by virtue of its superior soft tissue contrast with no ionizing radiation (3). Apparent diffusion coefficient (ADC) values and T2 mapping were used to evaluate TAO activity and to improve diagnostic accuracy. ADC values can reflect the movement of water molecules within the optic nerve (4). T2 mapping can be used to increase anatomic contrast and allowed accurate quantitatively measure the T2 relaxation time of the tissue (5). At present, the researches were mainly focused on the intraorbital optic nerve. However, the eye is just a part of the visual pathway. It is necessary to further study other parts of the visual pathway in TAO patients.

Diffusion tensor imaging (DTI), which offers quantitative data regarding the microstructural integrities of highly orientated tissues, is being used more often to assess different orbital illnesses, such as glaucoma, neuromyelitis optic spectrum disorders, and idiopathic optic neuritis (6–8). However, previous studies that applied DTI to assess microstructural changes in TAO patients are still limited, with most research focusing on the intraorbital optic nerve and orbital soft tissue (9–11). To our knowledge, there are very few studies on changes in the visual pathway in TAO patients (12). It has been proven the trans-synaptic degeneration (TSD) is a damage mechanism that occurred in the visual pathway (13). Hence, the pathology of the optic nerve may influence the optic chiasm, optic tract, optic radiation, lateral geniculate body, and visual cortex by TSD. Therefore, it is important to elucidate the changes in other parts of the visual pathway other than the intraorbital optic nerve.

The aim of the study was to compare the quantitative parameters of DTI in the visual pathway of TAO patients with different severities and to analyze correlations between clinical features and DTI quantitative parameters.

## Materials and methods

This study was approved by our institutional review board. All participants provided informed consent.

### Participants

The study enrolled 57 consecutive patients who were clinically diagnosed with TAO (14) and 30 healthy controls (HCs) from September 2018 to July 2021. According to the European Group on Graves' Orbitopathy (EUGOGO) (15), the research included 22 mild TAO patients (13 women and 9 men), 35 moderate-severe TAO patients (20 women and 15 men), and 30 HCs (20 women and 10 men). The exclusion criteria were as follows: (1) ophthalmopathies such as glaucoma and diabetic retinopathy; (2) undergone eyelid surgery, radiation therapy, decompression, or strabismus surgery; (3) inadequate image quality; (4) systemic disease history or psychiatric disorders.

### Clinical assessments

Laboratory measurements included: free thyroxine (FT4), free triiodothyronine (FT3), thyroid stimulating hormone (TSH), thyroglobulin antibody (TGAb), thyroid peroxidase (TPO), and thyrotrophin receptor antibody (TRAb). The following clinical variables were investigated: age, sex, visual acuity, exophthalmometry, intraocular pressure, smoking history, antithyroid treatment history, and clinical activity score (CAS) (16). Disease activity for each unit of the eye was evaluated according to the modified 7-item CAS. If CAS ≥ 3, the eye was defined as active phase, otherwise inactive (CAS < 3). Finally, a total of 58 eyes were defined as active and 56 eyes were defined as inactive.

### Image acquisition

Magnetic resonance examinations used a Siemens Prisma 3.0T MR scanner and 64-channel head coil. Participants were asked to close their eyes during MRI scanning. To reduce image artifacts caused by head movement, sponge pads were affixed to both sides of the head. Conventional imaging protocols included axial and coronal T1-weighted imaging (T1WI) and T2-weighted imaging (T2WI). The following were the scan parameters for the T1WI sagittal 3D thin-layer imaging: echo time (TE) 2.98 ms, repetition time (TR), 2,530 ms; acquisition matrix 256 × 256; slice thickness 1 mm; slice spacing 0, matrix (field of view, FOV) 256 mm × 256 mm. DTI was acquired based on a T1WI sagittal 3D thin-layer scan with the following imaging parameters: FOV, 224 mm × 224 mm; TR, 3,500 ms; TE, 63 ms; acquisition matrix, 200 × 200; layer thickness, 2.0 mm; layer spacing 0, total number of layers 75, flip angle, 90°; number of diffusion gradient directions (NDGDs), 64; *b* = 0 and 1,000 s/mm^2^. The scan location line was parallel to the intraorbital optic nerve.

**Figure 1:**
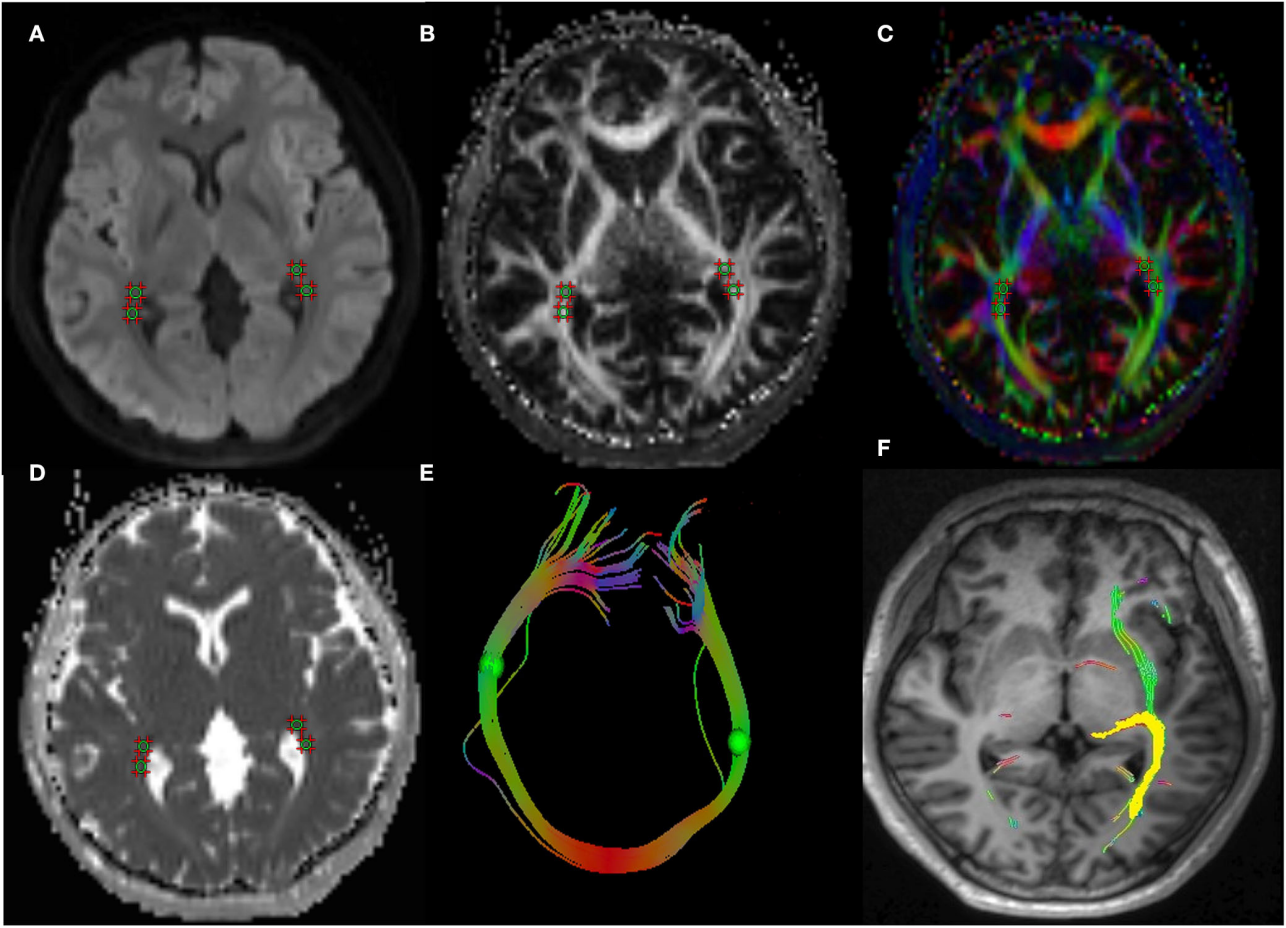
A 43-year-old woman with TAO. Schematic diagrams for measurements of visual pathway on DTI. Figure **(A–D)** showed the axial TRACEW, FA maps, color FA, and ADC. **(E)** showed an “open-loop structure” consisting of bilateral optic radiation white matter fibers and splenium of corpus callosum. **(F)** showed the visual pathway white matter fiber tractography, the yellow stands for the track result of LG, it shows the subcortical pathway reaching primary visual cortex through the optic radiation. A circle region of interest was manually set on LG and OR, with careful avoidance of surrounding tissues. Fiber tractography was then carried out to confirm correct placement of ROI. TAO: thyroid-associated ophthalmopathy, DTI: diffusion tensor imaging, FA: fractional anisotropy, ADC: apparent diffusion coefficient, LG: lateral geniculate, OR: optic radiation.

The study applied the Syngo.Via post-processing software equipped with a Siemens Prisma 3.0T post-processing workstation to measure the DTI parameters of visual pathway. The radiologist opened the TRACEW, FA, and apparent diffusion coefficient (ADC) images in MR Basic and altered TRACEW to show the LG and optic radiation (OR) at the clearest level. The region of interest (ROIs) was drawn at the bilateral LG and OR (with the TRACEW images as reference), and the diameter of each ROI was fixed at 5 mm. The FA and MD of the lateral geniculate and optic radiation were automatically computed by the software. In order to ensure the accuracy of the manually drawn ROI, we sketched the ROI anatomical site on T1WImprage continuous tracking method was used to construct fibers, and the fiber tracking began at the center of each voxel with an FA value >0.2 and ended at voxels with FA < 0.3 or the tract turning angles between two eigenvectors to be connected by the tracking were greater than 70. Then the software will automatically track and form a white matter fiber map associated with the outlined ROI. If the image is a white matter fiber map of visual pathway, it is considered that the ROI sketch is valid (Figure 1). The ROI was independently drawn by two experienced neuroradiologists who were unaware of the clinical conditions of the participants. The inter-observer agreement was calculated using the measurement results of these two observers. All DTI parameters were taken as the average value of the measurement results of the two radiologists.

### Statistical analysis

The statistical analysis was performed with SPSS 25.0 software. Numerical data are reported as mean ± standard deviation. The distribution of numerical variables was evaluated using the Kolmogorov–Smirnov test. The CAS is given as a median with an interquartile range (IQR) since it does not follow a normal distribution. One-way ANOVA was used to compare age-related differences, and the chi-squared test was applied to compare differences regarding gender and medication categories, the Fisher's exact test was performed to compare differences in smoking history. Differences in serum thyroid biochemical levels (TSH, FT3, FT4, TGAb, TRAb, and TPO) were analyzed using the independent-samples T test. Both TAO patients' eyes had the same severity level. Paired-samples T test was used to compare each patient's left/right eye characteristics (*p* > 0.05). The average values of each parameter type, such as FA and MD, were calculated. The Kruskal–Wallis test was performed to compare CAS between groups. One-way ANOVA was applied to compare intergroup differences, with pairwise comparisons between groups based on the least significant difference (LSD); then differences in DTI parameters of the LG and OR were compared between normal, mild, and moderate-severe groups. Pearson correlation analysis was conducted for the correlation analyses between DTI parameters and clinical variables. Meanwhile, Pearson correlation analysis was also analyzed the correlation between DTI parameters of LG and OR. In the multivariate analysis, all non-correlated variables were subjected to binary logistic regression to identify variables that can significantly diagnose TAO by using a forced-entry process. The odds ratios and 95% confidence intervals (*CIs*) were calculated.

The intraclass correlation coefficient (ICC) was calculated to evaluate the consistency in the DTI parameters reported by the two radiologists. A two-way ICC with random rate assumption was conducted. The results were interpreted as follows: < 0.40, poor; 0.40–0.60, moderate; 0.61–0.80, good; ≥0.81, excellent. The threshold of statistical significance was set at 0.05.

## Results

Table 1 provides an overview of the clinical and demographic characteristics of the 30 healthy controls and 57 TAO patients. There were no significant differences in the sex distribution, age or smoking habits between TAO patients and HCs (*p* > 0.05). Differences in the antithyroid treatment history and serum thyroid biochemical levels (TSH, FT3, FT4, TGAb, TRAb, and TPO) between mild and moderate-severe TAO patients were not significant (*p* > 0.05).

**Table 1:** Demographics of the healthy controls and thyroid-associated orbitopathy (TAO) patients.

	**Healthy controls** **(*n* = 30)**	**TAO patients** **(*n* = 57)**		***F*/*X*^2^**	***T* value**	***P*-value**
		**Mild** **(*n* = 22)**	**Moderate to severe** **(*n* = 35)**			
Age	41.5 ± 13.8	41.1 ± 12.6	42.0 ± 14.2	-	-	0.9681^a^
Gender (M/F)	10:20	9:13	15:20	0.657	-	0.7202^b^
Antithyroid medication, %(*n*)	-	54.5% (12)	57.1% (20)	0.037	-	0.8472^b^
Smoking history, %(*n*)	23.3% (7)	13.6% (3)	17.1% (6)	-	-	0.7343^c^
TSH (mIU/L)	-	2.71 ± 2.01	3.62 ± 2.73	-	−1.346	0.1844^d^
FT3 (pmol/L)	-	4.44 ± 1.59	4.46 ± 1.72	-	−0.050	0.9614^d^
FT4 (pmol/L)	-	14.62 ± 3.77	15.38 ± 4.86	-	−0.625	0.535^d^
TGAb (IU/mL)	-	56.46 ± 28.13	58.48 ± 27.03	-	0.250	0.8094^d^
TRAb (IU/L)	-	5.20 ± 2.52	6.47 ± 2.51	-	−1.858	0.0694^d^
TPO (IU/L)	-	45.59 ± 19.11	51.88 ± 17.94	-	−1.258	0.2144^d^

Numeric data are reported as means ± standard deviations. Statistical significance was indicated by p-values < 0.05. F, female; M = male; TAO, thyroid-associated orbitopathy, TSH, thyroid-stimulating hormone; FT3, free triiodothyronine; FT4, free thyroxine; TGAb, thyroglobulin antibody; TRAb, thyrotrophin receptor antibody; TPO, thyroid peroxidase. a: One-way ANOVA. b: Chi-squared test. c: Fisher's exact test. d: Independent-samples T test.

There was no statistically significant difference in the clinical characteristics (intraocular pressure, CAS, exophthalmos, or visual acuity) or DTI quantitative parameters of the left and right eyes of the subjects. The clinical variables for the mild and moderate-severe patients were as follows: average degrees of exophthalmos, 18.23 ± 2.86 and 19.67 ± 2.98 mm, average CAS, 2.00 (1.00, 2.00) and 4.00 (3.00, 5.00), average intraocular pressures, 17.86 ± 3.06 and 18.23 ± 3.93 mmHg, and average visual acuity, 0.95 ± 0.12 and 0.93 ± 0.14 (Table 2).

**Table 2:** Comparison of left and right visual pathway and clinical parameters.

**Parameters**		**Right eye**	**Left eye**	**Mean value**	***T* value**	***P*-value**
Lateral geniculate	FA	0.424 ± 0.035	0.424 ± 0.035	0.424 ± 0.035	1.008	0.318
	MD (× 10^−3^mm^2^/s)	0.965 ± 0.127	0.965 ± 0.126	0.965 ± 0.126	0.018	0.985
Optic radiation	FA	0.534 ± 0.048	0.534 ± 0.049	0.534 ± 0.048	1.370	0.176
	MD (× 10^−3^mm^2^/s)	0.882 ± 0.009	0.890 ± 0.175	0.886 ± 0.092	1.084	0.283
Exophthalmometry (mm)	Mild	17.86 ± 3.08	18.59 ± 3.32	18.23 ± 2.86	1.000	0.201
	Moderate –severe	19.60 ± 2.87	19.73 ± 3.56	19.67 ± 2.98	−0.304	0.763
CAS	Mild	2.00 (1.00,2.00)	1.00 (1.00,2.00)	2.00 (1.00,2.00)	−0.376	0.707
	Moderate-severe	4.00 (3.00,5.00)	4.00(3.00,5.00)	4.00 (3.00,5.00)	−0.615	0.538
Intraocular pressure (mmHg)	Mild	17.55 ± 3.31	18.18 ± 3.33	17.86 ± 3.06	−1.374	0.184
	Moderate-severe	18.31 ± 3.81	18.14 ± 4.28	18.23 ± 3.93	0.502	0.619
Visual acuity	Mild	0.95 ± 0.12	0.95 ± 0.16	0.95 ± 0.12	−0.271	0.789
	Moderate-severe	0.95 ± 0.14	0.92 ± 0.18	0.93 ± 0.14	1.234	0.226

Numeric data are reported as means ± standard deviations. CAS are reported as median (interquartile range). Statistical significance was indicated by p-values < 0.05. FA, fractional anisotropy; MD, mean diffusivity; CAS, clinical activity score.

**Figure 2:**
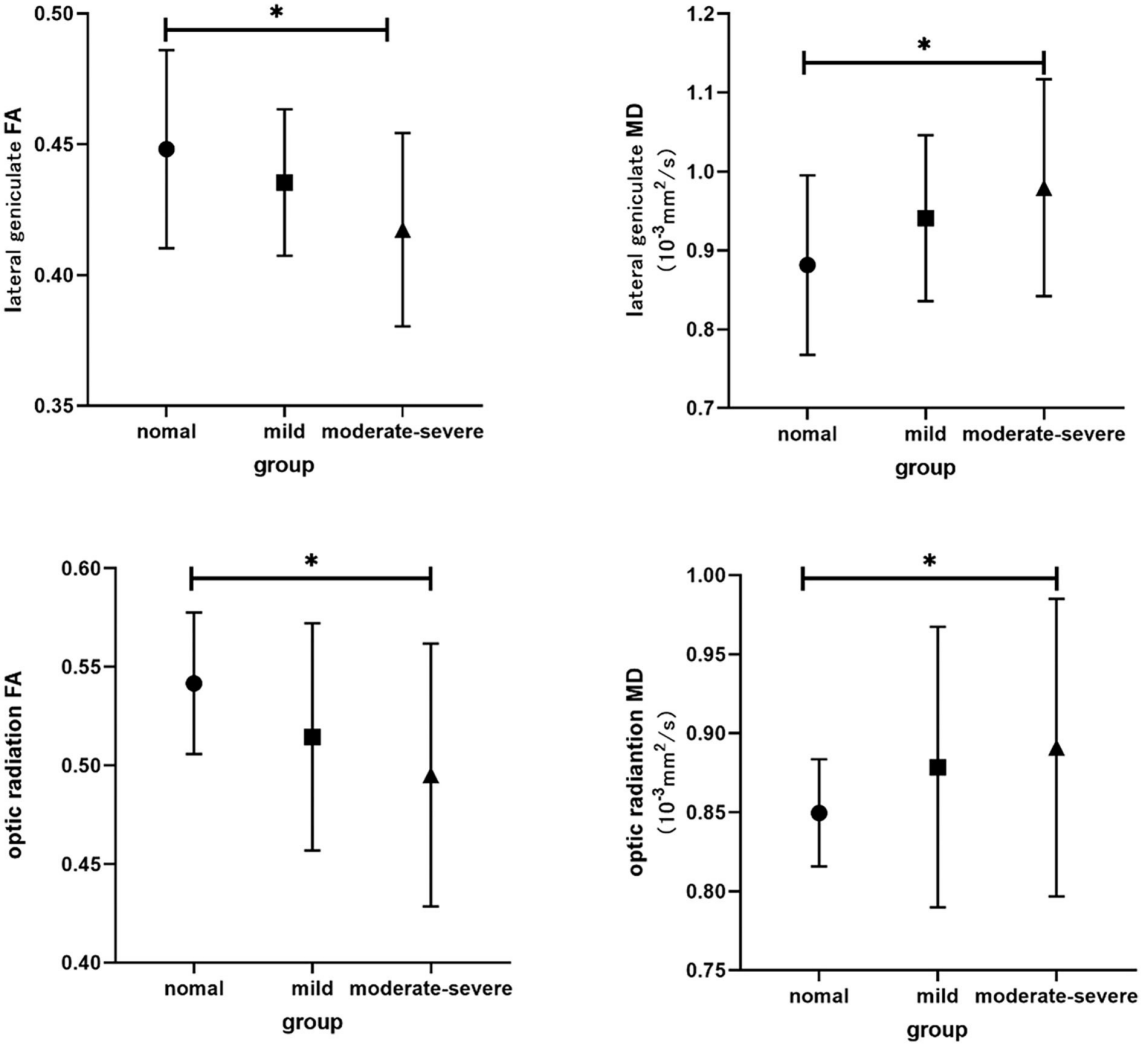
Bar graphs show comparisons of DTI parameters in visual pathway between groups. The main bars represent the mean value of the parameters and the error bars stand for standard deviation. The asterisk indicates a significant difference.

The interobserver agreement for quantitative imaging parameters between the radiologists demonstrated excellent agreement (ICC range, 0.793–0.915). Comparisons of the MRI-derived metrics are presented in Table 3. Moderate-severe TAO patients showed significantly lower FA and higher MD than HCs (all *p* < 0.01) in both the LG and OR (Figure 2).

**Table 3:** Diffusion tensor imaging parameters of the visual pathway in the healthy control group (HCs), mild and moderate-severe TAO groups.

**Parameter**		**HCs**	**Mild**	**Moderate-severe**	***F* value**	***P*-value**
Lateral geniculate	DTI-FA	0.448 ± 0.038	0.435 ± 0.028	0.417 ± 0.037	6.266	**0.003**
	DTI-MD (× 10^−3^mm^2^/s)	0.881 ± 0.114	0.941 ± 0.105	0.979 ± 0.138	5.236	**0.007**
Optic radiation	DTI-FA	0.450 ± 0.032	0.435 ± 0.028	0.417 ± 0.037	7.877	**0.001**
	DTI-MD (× 10^−3^mm^2^/s)	0.850 ± 0.034	0.880 ± 0.094	0.891 ± 0.094	2.379	**0.099**

Numeric data are reported as means ± standard deviations. Statistical significance was indicated by p-values < 0.05. FA, fractional anisotropy; MD, mean diffusivity, HCs, healthy control group, TAO, thyroid associated ophthalmopathy.

Normal compared to Mild: LG-FA, LG-MD, OR-FA, OR-MD. P > 0.05.

Normal compared to Moderate-to-Severe: LG-FA P = 0.001, LG-MD P = 0.002, OR-FA P < 0.001, OR-MD P = 0.034.

Mild compared to Moderate to Severe: LG-FA, LG-MD, OR-FA, OR-MD. P > 0.05.

*P* values < 0.05 were marked bold.

**Figure 3:**
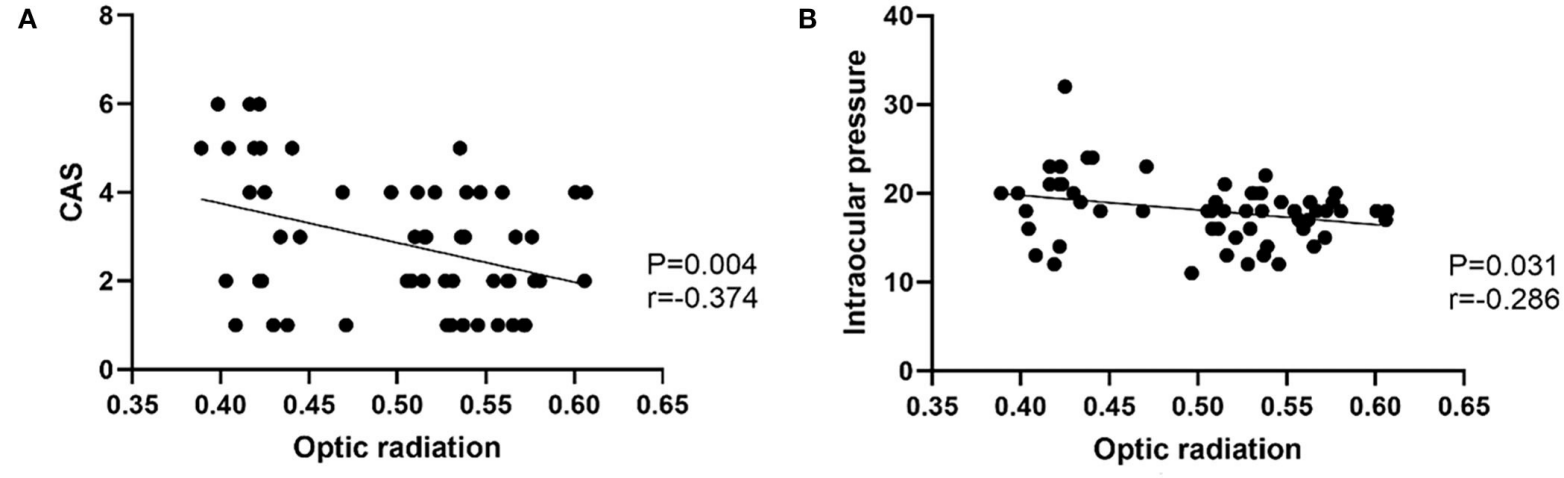
**(A)** Scatter diagrams of the correlations between optic radiation and CAS. **(B)** Scatter diagrams of the correlations between optic radiation and Intraocular pressure.

In the TAO group, FA of OR was negatively correlated with CAS (*r* = −0.374, *p* = 0.004), intraocular pressure (*r* = −0.286, *p* = 0.031) (Figure 3). There were no significant correlations between LG DTI parameters of visual pathway and clinical characteristics (intraocular pressure, CAS, exophthalmos, or visual acuity) (*p* > 0.05). Multivariate analysis showed that FA of LG and FA of OR were significant predictors of TAO (*P* = 0.015, *P* = 0.010) (Table 4).

**Table 4:** Logistic regression analysis of factors associated with TAO.

**Parameters**	**B**	**Stand error**	***P*-value**	**Exp(B)**	**95% CI for B**	
					**Lower bound**	**Upper bound**
Lateral geniculate FA	−0.016	0.007	**0.015**	0.984	0.971	0.997
Optic radiation FA	−0.014	0.005	**0.010**	0.986	0.976	0.997

B, regression correlation coefficient; CI, confidence interval; TAO, thyroid associated ophthalmopathy; FA, fractional anisotropy, MD, mean diffusivity. *P* values < 0.05 were marked bold.

## Discussion

Our study sought to investigate the potential of DTI in characterizing the pathological alterations of LG and OR. There were three main findings. First, moderate-severe TAO patients had significantly lower FA and higher MD in the LG and OR compared with HCs. Second, FA of OR was negatively correlated with CAS and intraocular pressure. Third, FA of LG was a significant predictor of TAO (*p* = 0.008).

Thyroid-associated ophthalmopathy is an autoimmune inflammatory orbital disorder, the disease usually involves the extraocular muscles and orbital fat (18). The disease's immunological foundation is characterized by perivascular and diffuse infiltration of CD4+ and CD8+ T cells, B cells, plasma cells, and macrophages (19). Dysthyroid optic neuropathy is the most severe clinical stage of TAO, and a prior study found that approximately 5% of TAO patients developed progressive optic neuropathy (20). Several hypotheses on the pathophysiology of DON have been proposed (2). The most widely accepted explanation is that the apical optic nerve is directly compressed by the enlarged extraocular muscle (EOM), reducing axoplasmic flow. In a small proportion of patients, orbital imaging reveals substantial proptosis with stretching of the optic nerve rather than minimal or no apical crowding. Since all nerves have a threshold tensile strength beyond which axons or blood supplies may shear, this process can also lead to optic nerve injury. Our previous research found (17) that DTI parameters can reflect changes in the intraorbital optic nerve before dysthyroid optic neuropathy in TAO patients.

In the visual pathway, trans-synaptic degeneration (TSD) has been shown to be a damaging mechanism (13, 21). According to this theory, through anterograde and retrograde directional TSD, intraorbital optic nerve disease may have an impact on the optic chiasm, optic tract, optic radiation, LG body, and visual cortex. Recent studies have indicated that (22–24) TAO can cause morphological and microstructural abnormalities in the brain region associated with the visual pathway (occipital lobe). All this evidence suggests that TAO is an eye-brain neuropathy that is not limited to damage to the eye. A variety of radiologic metrics has been utilized to assist in the diagnosis of optic nerve alterations, such as detection of apical crowding on scanning, decreased diameter of the optic nerve on T1-weighted imaging, ADC value to measure the optic nerve, and T2 mapping to detect intraorbital optic nerve changes (25, 26), study on the posterior visual pathway still remains few.

Diffusion tensor imaging is a noninvasive functional magnetic resonance technique (27, 28). It can examine white matter structure *in vivo* since the brain's white matter tracts are made up of bundles of axons that are often arranged in a variety of orientations. The anatomy of the optic nerve is similar to that of the cranial nerves. DTI has, therefore, lately been used to assess the optic nerve (29, 30). Several previous studies (11, 31) have suggested that DTI parameters are correlated with the clinical course of TAO. However, current research on the TAO visual pathway is mainly limited to the intraorbital optic nerve, and there is very little research on other parts of the optic pathway. Liu et al. (12) evaluated the microstructural changes along the whole visual pathway in TAO patients using DTI. The result of our findings is consistent with the findings of Liu et al.: the visual pathway FA and MD values are associated with disease severity. As the disease severity increased, there was a tendency of gradually decreased FA and increased MD. In addition, our study also performed DTI measurements on the lateral geniculate body. The results of our study suggest a significant decrease in LG-FA and an obvious increase in LG-MD. DTI can be used to measure the movement of water molecules perpendicular and parallel to axonal tracts (7). The most widely accepted mechanisms of injury to the intraorbital optic nerve in TAO are mechanical compression and ischemia. Extensive damage caused by myelin and axon loss reduces anisotropy. This leads to increased diffusion perpendicular to the white matter tract, enhanced overall diffusivity (MD), and decreased tissue directionality (FA) (27). Neuronal and axonal degeneration following injury to cells with which they synapse is called trans-synaptic degeneration (32). Hence, the pathology of the intraorbital optic nerve may affect the LG, OR, and even visual cortex by both anterograde and retrograde directional TSD. This research indicates that DTI might show the visual pathway injury in TAO. Especially in patients who have not yet developed DON, our study found that moderate-severe patients have developed microstructural changes in the visual pathway. The study results are helpful for the clinical management and treatment of patients, and for preventing the occurrence of DON.

In our study, a significant negative correlation between FA of OR and CAS, IOP were explored in the patient cohort. The correlation results further demonstrated the possibility of utilizing DTI to assess visual pathway changes in TAO, showing that disruptions in the visual pathway progress along with increased inflammation and crowding of the orbital tissues. As previous research stated (12), OR–MD showed no significant correlation with any of the clinical variables, which were consistent with our study. While the OR–FA was positively correlated with VA and IOP, which is in direct contrast to our findings. A possible reason for this discrepancy might be the composition of TAO patients, the prior study has included some DONs, which may have affected the outcome analysis.

The study has several limitations. First, the sample size for our investigation was small. Second, DON patients were not included in our current study, as it is known to us, DON (33) is a severe, sight-threatening complication of TAO. We believe that the changes in DTI and CBF in the visual pathway of DON patients will be more obvious. Third, this research did not follow up the treated TAO patients with MRI. A complete follow-up MRI examination would help us to better understand TAO disease and the patient's response to treatment. Therefore, in the future, we will enroll more TAO patients at different disease stages, and conduct follow-up observation after treatment.

## Data availability statement

The original contributions presented in the study are included in the article/supplementary material, further inquiries can be directed to the corresponding authors.

## Ethics statement

The studies involving human participants were reviewed and approved by Medical Ethics Committee of Beijing Friendship Hospital. The patients/participants provided their written informed consent to participate in this study.

## Author contributions

Conception and design: RL, JL, and ZW. Administrative support: ZW and JL. Provision of study materials or patients and Collection and assembly of data: RL. Data analysis and interpretation: RL and JL. All authors contributed to the article and approved the submitted version.

## Conflict of interest

The authors declare that the research was conducted in the absence of any commercial or financial relationships that could be construed as a potential conflict of interest.

## Publisher's note

All claims expressed in this article are solely those of the authors and do not necessarily represent those of their affiliated organizations, or those of the publisher, the editors and the reviewers. Any product that may be evaluated in this article, or claim that may be made by its manufacturer, is not guaranteed or endorsed by the publisher.
